# Probing Synergistic Targets by Natural Compounds for Hepatocellular Carcinoma

**DOI:** 10.3389/fcell.2021.715762

**Published:** 2021-07-28

**Authors:** Jian Gao, Zuojing Yin, Zhuanbin Wu, Zhen Sheng, Chao Ma, Rui Chen, Xiongwen Zhang, Kailin Tang, Jian Fei, Zhiwei Cao

**Affiliations:** ^1^Department of Gastroenterology, School of Life Sciences and Technology, Shanghai Tenth People’s Hospital, Tongji University, Shanghai, China; ^2^Shanghai Model Organisms Center, Inc., Shanghai, China; ^3^Shanghai Engineering Research Center of Molecular Therapeutics and New Drug Development, School of Chemistry and Molecular Engineering, East China Normal University, Shanghai, China

**Keywords:** synergistic targets, natural compounds, drug discovery, hepatocellular carcinoma, cancer system biology

## Abstract

**Background:**

Designing combination drugs for malignant cancers has been restricted due to the scarcity of synergy-medicated targets, while some natural compounds have demonstrated potential to enhance anticancer effects.

**Methods:**

We here explored the feasibility of probing synergy-mediated targets by Berberine (BER) and Evodiamine (EVO) in hepatocellular carcinoma (HCC). Using the genomics-derived HCC signaling networks of compound treatment, NF-κB and c-JUN were inferred as key responding elements with transcriptional activity coinhibited during the synergistic cytotoxicity induction in BEL-7402 cells. Then, selective coinhibitors of NF-κB and c-JUN were tested demonstrating similar synergistic antiproliferation activity.

**Results:**

Consistent with *in vivo* experiments of zebrafish, coinhibitors were found to significantly reduce tumor growth by 79% and metastasis by 96% compared to blank control, accompanied by anti-angiogenic activity. In an analysis of 365 HCC individuals, the low expression group showed significantly lower malignancies and better prognosis, with the median survival time increased from 67 to 213%, compared to the rest of the groups.

**Conclusion:**

Together, NF-κB and c-JUN were identified as promising synergistic inducers in developing anti-HCC therapies. Also, our method may provide a feasible strategy to explore new targeting space from natural compounds, opening opportunities for the rational design of combinational formulations in combatting malignant cancers.

## Introduction

Characterized by high heterogeneity, rapid development, and early metastasis, hepatocellular carcinoma (HCC) has been referred to as the leading cause of cancer-related death worldwide with the majority of cases found in the Asia-Pacific region (Yang et al., 2019). Many cellular pathways were reported to be involved in the induction and maintenance of HCC, such as proliferation, angiogenesis, aberrant metabolism, autocrine growth factors, hypoxia, and so on (Bayat Mokhtari et al., 2017). As of now, only a few inhibitors of sorafenib, lenvatinib (first-line), and regorafenib (second-line) were clinically approved for HCC treatment targeting the VEGFR (vascular endothelial growth factor receptor) (Llovet et al., 2016; Villanueva, 2019). Meanwhile, the PD-1 checkpoint inhibitor nivolumab was reported with conditional approval for sorafenib-treated patients in the United States (Huppert et al., 2020). Being usually diagnosed at an advanced stage, HCC patients clinically receive limited survival benefits from single-drug treatments due to the adaptive therapeutic resistance rapidly developed (Tagliamonte et al., 2020). While conventional chemotherapy was frequently hesitated to recommend, the 5-year survival rate of HCC was reported to be only 18% (Villanueva, 2019).

To seek more effective clinical benefits, combinational agents have been increasingly explored for HCC by simultaneously targeting multiple deregulated targets/pathways, particularly those with potential to produce synergistic therapeutic benefits (Frei et al., 1965). For instance, by blocking both the Ras/Raf/MAPK and PI3K/AKT/mTOR pathways, PI-103, a potent inhibitor of AKT (Ser473) phosphorylation, combined with sorafenib was reported to synergistically inhibit epidermal growth factor (EGF)-stimulated HCC Huh7 cell proliferation (Gedaly et al., 2010). Also, by simultaneously targeting the YAP and EGFR signaling pathways, a strong synergistic effect of simvastatin and gefitinib was detected in BEL-7402 HCC cells (Xia et al., 2018). In this direction, large-scale screening has been initiated by CRISPR-Cas9 technology in searching for additional targets whose knockout can synergize sorafenib efficacy (Wang et al., 2018). Also, computational models have extensively been set up to predict anticancer synergy among candidate drugs with the targeting network and genomic profiling being obtained (Sun et al., 2015).

Notably, an active area of interest is the inclusion of natural therapeutic compounds to sensitize available drugs or formulate new combinations, considering the paucity of FDA-approved agents for HCC. Compound Kushen injection, an extraction from the roots of *Sophora flavescens* and *Smilax glabra* Roxb, was investigated as a sensitizer to sorafenib through relieving immunosuppression in orthotopic, subcutaneous, postsurgical recurrence and tumor rechallenge models (Yang et al., 2020). Moreover, resveratrol, a phytoalexin derived from plants with antioxidant and chemopreventive effects, was reported as a synergistic partner with etoposide in inhibiting proliferation of the HCT-116 and HepG2 cell lines (Amiri et al., 2013). More importantly, a number of natural compounds have been reported with promising anti-HCC effects through *in vitro* or *in vivo* experiments. For instance, Berberine (BER), the main alkaloid of *Coptis chinensis*, was found to inhibit the growth of HepG2 cells by promoting apoptosis through the NF-κB p65 pathway and suppressing glutamine uptake (Li et al., 2017; Zhang et al., 2019). Evodiamine (EVO), an active alkaloid from *Evodia rutaecarpa*, also induced Akt-mediated apoptosis in HepG2 cells (Yang et al., 2017). EVO alone could inhibit the proliferation and promote the apoptosis of HepG2 and BEL-7402 cells *in vitro* and attenuate xenograft tumor formation in nude mice *in vivo* (Zhao et al., 2020). When combined, BER and EVO (CBE) were shown to demonstrate synergistic effects in inducing apoptosis and cell cycle arrest in the HCC cell line of SMMC-7721 (Wang et al., 2008).

Apart from using them for guiding the development of multicomponent therapies, these natural compounds with potential anticancer effects may be explored as valuable probes to detect combinational inducers in targeting space that operate synergistically through multiple signaling pathways. Then further design of selective inhibitors to modulate synergistic targets is therefore becoming an attractive possibility for the treatment of HCC disease. In view of recent synergy predictive efforts (Sun et al., 2015; Yang et al., 2015; Yan et al., 2020), such a task might be accomplished under the analysis of genomic profiling change, the relevant signaling networks, coupled with *in vitro* and *in vivo* validations. Here, this feasibility was evaluated by investigating synergy inducers by the cotreatment of BER and EVO in BEL-7402, a traditional Asian HCC cell line derived from a Chinese male with primary HCC.

## Materials and Methods

### Reagents

BER and EVO (purity: > 98%) were purchased from Shanghai Tauto Biotech Co (Shanghai, China). BER was dissolved in sterile saline, and EVO was dissolved in methyl sulfoxide (DMSO) at 0.01 mol/L to make the stock solution. Other chemicals, except where especially noted, were purchased from Sigma-Aldrich Chemical (St. Louis, MO, United States).

### Viability and Synergy Evaluation

Dose-dependent cytotoxicity was measured by the MTT assay as described previously. Briefly, the BEL-7402 cells were seeded in 96-well flat-bottomed plates (Corning, Acton, MA, United States) at a density of 4 × 10^3^ cells/well in a complete medium for 12 h. Then, BER and/or EVO were added alone or in combination at indicated concentrations and left in contact for 48 h. At the end of the incubation, 20 μl MTT (5 mg/ml) was added into each well, and the plates were incubated at 37°C for 3 h. Then 50 μl lysis buffer [20% SDS in 50% N, N-dimethylformamide, containing 0.5% (v/v) 80% acetic acid and 0.4% (v/v) 1 mol/L HCl)] was added into each well and incubated for 12 h. After that, the optical density (proportional to the number of live cells) was assessed with a Genios Microplate Reader (Tecan, Research Triangle Park, NC, United States) at 570 nm.

### cDNA Library Preparation and RNA-Seq

For the extraction of total RNA, cells were first rinsed in PBS and then lysed in a Trizol reagent (Invitrogen, United States). Then, mRNA selection, cDNA library preparation, and sequencing were performed by Shanghai Center for Bioinformation Technology (SCBIT) on an Illumina Hiseq 2000 sequencing platform according to the specifications of the manufacturer. Briefly, mRNA was selected using oligo(dT) probes and then fragmented using divalent cations. cDNA was synthesized using random primers, modified and enriched for attachment to the Illumina flow cell. The raw Illumina sequencing data generated in this study have been deposited as a series with the accession number SRP066753 in the NCBI Sequence Read Archive database (SRA).

### Analysis of the RNA-Seq Data

The raw reads were cleaned by removing adaptor sequences, empty reads, and low-quality sequences (reads with unknown sequences “N” or less than 25 bp). Read pairs with at least 36 bp left in each read were kept as clean reads. Then, clean reads were mapped to human reference genome (Ensembl GRCh37.66 = hg19) using aligner Tophat (Trapnell et al., 2012) (version 2.0.6) with parameters (-r/–inner-mate-distance: 70, -g/–max-multihits: 1, –no-coverage-search, –no-novel-junc). Based on the alignment output from TopHat, cuffdiff, an add-on package in Cufflinks, was used to assess the difference in gene expression after those treatments. The differentially expressed genes were screened with the general criteria [an absolute log2 (fold change) ≥ 1].

### The Signal Reporter Assay for AP-1/NF-κB

In brief, cells were transfected with the reporter or control constructs by using an Attractene transfection reagent according to the protocol of the manufacturer (Qiagen, Inc., Valencia, CA). After 24-h transfection, cells were exposed to BER (57.36 μM), EVO (4.68 μM), or the combination of BER and EVO (15.78 and 1.58 μM, respectively) for 12 h, and then Luciferase assays were measured using the Dual-Luciferase Reporter assay (Promega, United States) according to the protocol of the manufacturer. Firefly luciferase activity was normalized against Renilla luciferase activity.

### Cell Culture

The human hepatocellular carcinoma BEL-7402 cells (Cat. No.: TCHu_10) were obtained from the cell bank of the Institute of Biochemistry and Cell Biology, Shanghai Institutes for Biological Sciences, Chinese Academy of Sciences.^1^ Cells were maintained in a humidified 37°C atmosphere containing 5% CO_2_ and cultured in an RPMI-1640 medium (GIBCO, Grand Island, NY, United States) supplemented with 10% fetal bovine serum (GIBCO), 2 mmol/L L-glutamine, 50 units/ml penicillin, and 50 mg/ml streptomycin.

### Validation of the Synergistic Effects of NF-κB and JNK *in vivo*

In this study, zebrafish embryos containing two strains named of “fli1a-EGFP” and “Casper Casper” were treated with JSH-23 and JNK-IN-8 as well as 0.1% DMSO as vehicle control in 2 days postfertilization (dpf) -5dpf with 20 models in each condition to determine the toxicity and safety. All compounds were dissolved in 0.1% DMSO (in fish water).

After that, DiI-stained human BEL-7402 cells were successfully grafted into the yolk sac of zebrafish embryo at 2 dpf without immunosuppressant treatment. And approximately 200 cells were injected into the yolk sac and assessed by fluorescence microscopy at the 0 days post-injection (dpi). Then, at the 5 dpi, CM-DiI red fluorescence dye-labeled BEL-7402 cancer cells were injected into the embryos.

### Proliferation and Survival Analysis of Patients From the TCGA Database

The FPKM values of NF-κB1 (the synonym of NF-κB), MAPK8 (the synonym of JNK), and Ki-67 related with proliferation, as well as the clinical survival status and overall survival (OS), were obtained from the human protein atlas database, which recorded the gene FPKM value and clinical information of patients from the TCGA database.

According to the best cutoff to divide different survival of NF-κB1 (6.18) and MAPK8 (3.06) recorded in this database, the patients were divided into the low or high expression of gene single or combination. Then, the KM survival curve was obtained on two types of samples by using the survival and survminer packages of the R language, and the survival difference was analyzed by the log-rank test. Besides, the boxplot Ki-67 expression among different types of samples was further accomplished by using the ggpubr package of the R language.

### Bioinformatics and Statistical Analysis

DAVID (the database for annotation, visualization, and integrated discovery) bioinformatics resources (Huang da et al., 2009) were used to explore the cellular pathways enriched by the differentially expressed genes. Those pathways with a *p*-value < 0.05 (modified Fisher’s exact test) and including at least two differentially expressed genes were considered significant.

Statistical analysis was determined using the two-sided Student’s *t*-test and ANOVA test. The criterion for statistical significance was established as a *p*-value of < 0.05.

## Results

### CBE Synergistically Inhibited BEL-7402 Proliferation With Transcriptomics Profiling Changed

**FIGURE 1:**
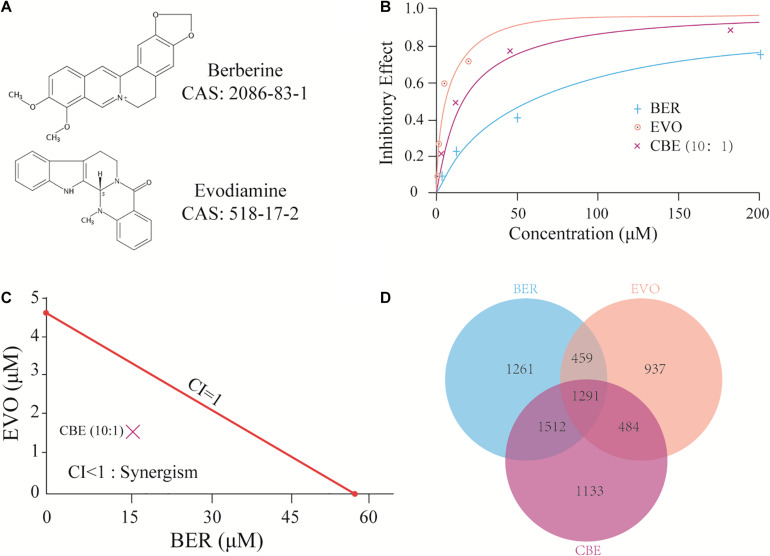
CBE synergisti cally inhibited the growth of BEL-7402 cells. **(A)** The chemical structure of BER and EVO. **(B)** Dose-response curves of BER, EVO, and CBE in BEL-7402 cells. Cells were treated with BER, EVO, or CBE for 48 h. To prepare CBE, a constant ratio (BER/EVO = 10:1) was used, and the curve was plotted against BER concentration. Cell viability was determined by MTT assay. **(C)** Isobologram for CBE at the dose ratio of 10:1. The red line represents the intercept line of the IC50 values for BER or EVO alone, and the “×” indicates the IC50 value for CBE at the dose ratio of 10:1. CI < 1, CI = 1, and CI > 1 indicate synergism, an additive effect, and antagonism, respectively. **(D)** The Venn plot of DEGs under the treatments of BER, EVO, and CBE.

Based on previous studies (Yang and Huang, 2013; Zhao et al., 2020), BER and EVO were illustrated as a pair of natural probes to evaluate the feasibility of identifying synergistic targets in BEL-7402. The chemical structures of BER and EVO are shown in Figure 1A. The cytotoxicity of BER, EVO, and their combination at a BER/EVO ratio of 10:1 was tested by the MTT method in BEL-7402 (see section “Materials and Methods”). As shown in the dose-response curves in Figure 1B, treatment with BER, EVO, or CBE inhibited the growth of BEL-7402 cells in a dose-dependent manner. The derived IC50 values at 48 h of treatment were 57.36 μM for BER and 4.68 μM for EVO, indicating that EVO might be more cytotoxic to BEL-7402 cells than BER. When BER and EVO were combined, generating CBE, their IC50 values were reduced to 15.78 μM for BER and 1.58 μM for EVO (Figure 1B). According to the combination index (CI) determined with the Chou–Talalay method (Ashton, 2015), the synergistic combination effect was attained at a constant molar ratio of BER/EVO = 10:1, which was approximately equal to the ratio of the IC50 values. The drug combination isobologram in Figure 1C shows that the CI value at this dose ratio was 0.61, suggesting the synergistic antiproliferative effect of CBE on BEL-7402 cells (CI < 1.0).

To investigate the potential synergy-medicated mechanism of CBE, whole-transcriptome profiling of BEL-7402 cells after 24 h of treatment with BER, EVO, or CBE was performed by RNA-seq to examine rapid changes in gene regulation (see section “Materials and Methods”). BER and EVO at the doses corresponding to their respective IC50 values were used to treat BEL-7402 cells (57.36 μM for BER, 4.68 μM for EVO, and 15.78 and 1.58 μM for BER and EVO, respectively, when combined in CBE). After analyses by TopHat and Cuffdiff (Trapnell et al., 2012), differentially expressed genes (DEGs) following treatment with BER, EVO, or CBE were detected (Supplementary Tables 1–3). As shown in Figure 1D, 29.21% (1291/4420) of DEGs induced by CBE treatment overlapped with those induced by both BER and EVO treatment. In addition, 25.63% (1133/4420) of the DEGs were uniquely induced by CBE, indicating their potential involvement in drug synergy.

### Synergy Responding Elements Suggested by HCC Signaling Transduction Network

**FIGURE 2:**
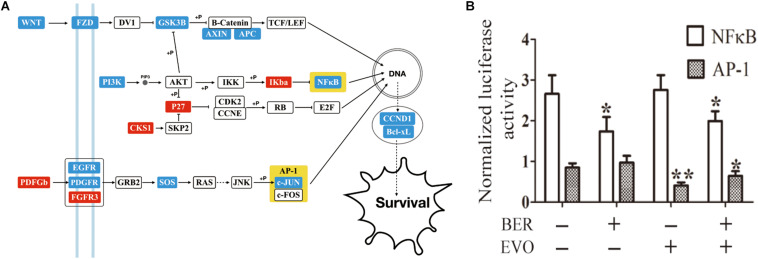
The detection of key players in CBE treatment based on differential signaling network. **(A)** Predicted signaling transduction network inferred for CBE treatment. Red boxes indicate upregulation, and blue boxes indicate downregulation under CBE treatment compared to blank control. Key points are highlighted in yellow. **(B)** Changes in the transcriptional activity of NF-κB and AP-1 by BER, EVO, or CBE treatment. BEL-7402 cells were transfected with the reporter construct for 24 h and then treated with BER, EVO, or CBE for 12 h. For each sample, firefly luciferase activity is presented as normalized to Renilla luciferase activity. The results from each group are presented as the mean ± SD of at least three samples. The drug dose was equal to that used the in the RNA-seq experiment. Columns, mean; bars, SD (*t*-test; *p < 0.05; **p < 0.01).

Pathway enrichment of the DEGs induced by CBE was obtained by the bioinformatics tool DAVID (Supplementary Table 4; Huang da et al., 2009) compared to blank control. When being overlapped with the significant cancer pathways (Sanchez-Vega et al., 2018), three enriched pathways were identified as canonical oncogenic pathways. An extended signal transduction network induced by CBE treatment was then reconstructed based on the cross-talks between canonical carcinogenic pathways (Sanchez-Vega et al., 2018) with DEGs highlighted in different colors compared to blank control (Figure 2A). As being suggested, cotreatment might downregulate multiple genes in the same pathway of PI3K, WNT, or MAPK, as well as genes in parallel pathways, such as NF-κB and c-JUN, which directly regulated the downstream biological process and thus inferred as key responding elements.

In addition to mRNA and transcriptional activity, the transcriptional activity of NF-κB1 and c-JUN (reporter gene in AP-1) was also found to be significantly reduced after 6-h incubation upon CBE treatment (Figure 2B, see section “Materials and Methods”). All the above results confirmed that CBE simultaneously suppressed the activation of the NF-κB and AP-1 complex. NF-κB and AP-1 are signaling located in the parallel cancer signaling of PI3K and MAPK pathways and are well known with multiple functions of cancer cell proliferation, migration, and invasion (Jiang et al., 2019). Based on previous empirical observation that targeting parallel intracellular signaling or membrane-downstream pathways tend to produce enhanced anticancer effects (Naoum et al., 2018), NF-κB and c-JUN were inferred as key responding elements inducing synergistic anti-HCC effects.

### Coinhibitors Synergistically Suppressed HCC Growth and Spreading *in vitro* and *in vivo*

**FIGURE 3:**
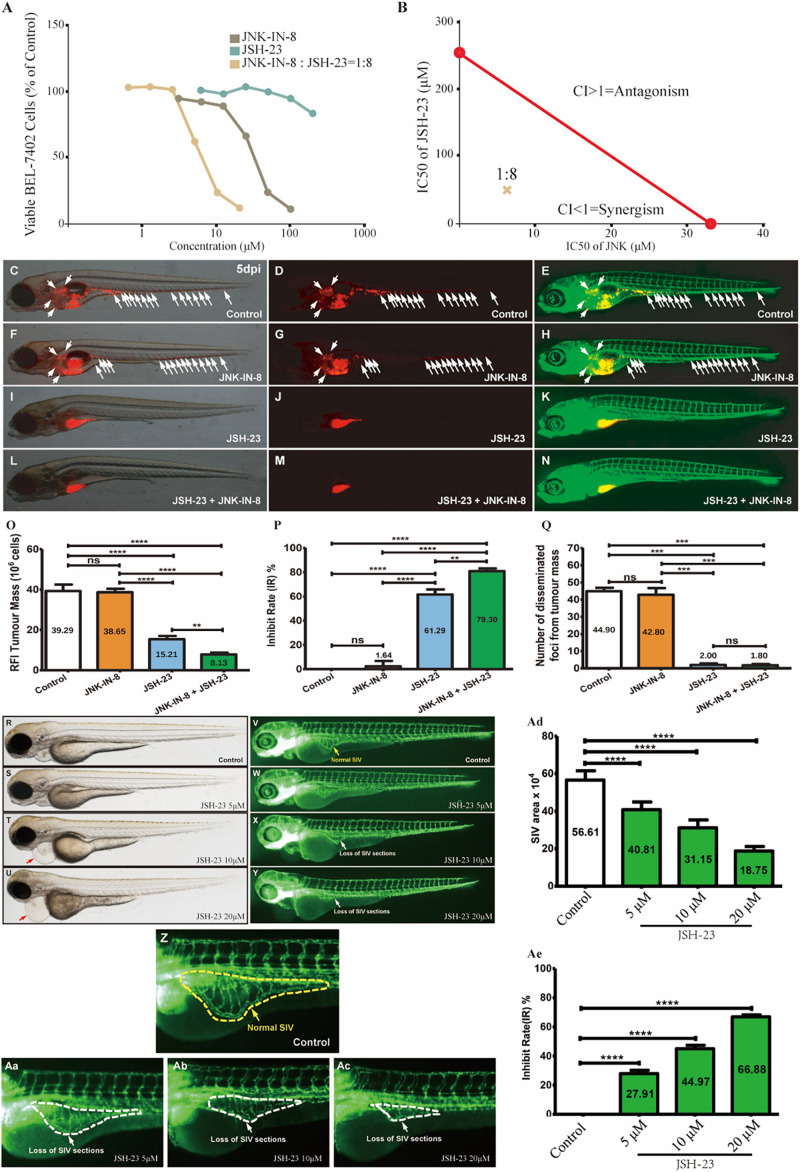
Coinhibition of NF-κB and c-JUN synergistically suppressed HCC growth and *in vitro* and *in vivo*. **(A)** The dose-response curves for each inhibitor alone or in combination. For the combination, the *x*-axis represents the concentration of JNK-IN-8. **(B)** Isobologram for the combination of JNK-IN-8 and JSH-23 in BEL-7402 cells. The red line represents the intercept line of the IC50 values for JNK-IN-8 and JSH-23 alone, and the “×” indicates the IC50 value for the two inhibitors at a dose ratio of 1:8. CI < 1, = 1, and > 1 indicate synergism, an additive effect, and antagonism, respectively. **(C–E**,**F–H**,**I–K**,**L–N)** Treatments administered to a zebrafish tumor xenograft model were vehicle control, JNK-IN-8 (10 μM) alone, JSH-23 (5 μM) alone, and JNK-IN-8 (10 μM) combined with JSH-23 (5 μM). BEL-7402 cells were labeled with CM-DiI red fluorescence dye, and white arrowheads indicate disseminated tumor foci in 5-dpi zebrafish embryos. **(O–Q)** Quantification of the tumor mass, the inhibition rate, and the number of disseminated tumor foci. **(R–Ac)** Representative bright field and fluorescent images of control embryos or embryos treated with JSH-23 at 4 days post-fertilization (dpf). In control embryos, SIVs developed as a smooth basket-like structure over the yolk at 4 dpf **(V,Z**, yellow dashed lines). In contrast, the treatment of embryos with JSH-23 resulted in specific defects in SIV formation **(W–Y,Aa–Ac**, white dashed lines). Treatment with 10 μM JSH-23 for 48 h caused pericardial edema (red arrow). The complications were caused by vascular defects in the SIV (white arrow). **(Ad,Ae)** Quantification of the SIV area shows a significant decrease in the SIV area in JSH-23-treated embryos compared to control embryos. RFI, relative fluorescence intensity; dpi, days post-injection. Columns, mean; bars, SEM (*n* = 10; ANOVA; **p < 0.01, ***p < 0.001, ****p < 0.0001).

To verify this hypothesis, JSH-23, a highly specific and commonly used NF-κB transcription activity inhibitor (Su et al., 2018), and JNK-IN-8, an irreversible pan-inhibitor of c-JUN N-terminal kinase (JNK) for JNK1, JNK2, and JNK4 (Lalaoui et al., 2016), were used alone or in combination to treat BEL-7402 cells *in vitro* for different durations (Figure 3 and Supplementary Figure 1, see section “Materials and Methods”). After 72 h, cotreatment at different dos ratios indicated that the best ratio of JNK-IN-8/JSH-23 in terms of maximizing their synergistic effect was 1:8 (Supplementary Figure 1). At this ratio, strong synergy was observed after 24 h of combination treatment, as illustrated in Figures 3A,B. The IC50 values were fitted as 33.36 μM for JNK-IN-8 and 255.95 μM for JSH-23. JSH-23 seemed not to suppress BEL-7402 cell proliferation alone. But when combined with JNK-IN-8, the IC50 values were determined to be 6.22 μM for JNK-IN-8 and 49.77 μM for JSH-23, with a CI index of 0.35. These results support the above hypothesis that coinhibition of NF-κB and c-JUN leads to a synergistic antiproliferative effect in BEL-7402 cells.

To further validate whether the coinhibition of NF-κB and c-JUN could also exert synergistic antitumor effects *in vivo*, we established a tumor metastasis model by using zebrafish embryos xenografted with BEL-7402 HCC cells (Supplementary Figure 2). To determine the safe doses of the inhibitors *in vivo*, zebrafish were treated from 2 days post-fertilization (dpf) to 5 dpf, and mortality was recorded every 24 h to derive the maximum non-lethal concentration (MNLC) and LC50 values of lead compounds. The MNLC and LC50 values in zebrafish were 9.3 and 20.1 μM, respectively, for JSH-23, and 24.4 and 56.2 μM, respectively, for JNK-IN-8 (Supplementary Figure 3). Detailed information regarding the evaluation of the safety of the combination treatment can be found in Supplementary Figure 3. Based on these results, we administered 5 μM JSH-23 and 10 μM JNK-IN-8 alone or in combination with BEL-7402 xenografted embryos at 2 days post-injection (dpi). As shown in Figures 3C–Q, inhibition of c-JUN alone had a non-significant anti-HCC effect but enhanced tumor sensitivity to JSH-23 compared to that in the vehicle-treated control, leading to an overall significant synergistic effect through reducing tumor mass by 79% (from 39.29 to 8.13) and disseminated tumor foci by 96% (from 44.90 to 1.80) when combined with JSH-23. In contrast to the poor *in vitro* antiproliferative activity, the NF-κB inhibitor JSH-23 showed striking anti-HCC effects in reducing both tumor mass by 61% (from 39.29 to 15.21) and disseminated tumor foci by 96% (from 44.90 to 2.00) when compared with the vehicle-treated control, which showed nearly no inhibition. In comparison with JSH-23 mono-treatment, combination treatment with JSH-23 and JNK-IN-8 reduced the average tumor mass by 46.55% from 15.21 to 8.13, showed an inhibition rate increased by 29.38% from 61.29 to 79.30%, and reduced the average number of disseminated tumor foci by 10.00% from 2 to 1.8. JSH-23 alone almost completely inhibited the dissemination of tumor foci, demonstrating its great antimetastatic potential. In summary, the above results indicate that the coinhibition of NF-κB and c-JUN can produce synergistic anti-HCC effects, with one mechanism (antimetastasis effects) contributing to the inhibition of NF-κB.

Interestingly, pericardial edema was also observed in zebrafish embryos after JSH-23 treatment (Supplementary Figure 4). Since pericardial edema is often caused by vascular defects in the sub-intestinal vein (SIV), the efficacy of JSH-23 was assessed in a zebrafish angiogenesis model by evaluating the SIV. As Figures 3R–Ae show, upon treatment with JSH-23 at the selected doses (5, 10, and 20 μM), the inhibition rate of the SIV area was 27.91, 44.97, and 66.88%, respectively, and increased in a dose-dependent manner. JSH-23 impaired the formation of the zebrafish SIV, demonstrating its ability to inhibit angiogenesis *in vivo*, in contrast with vehicle control treatment, which showed nearly no inhibition, or JNK-IN-8 treatment, which had no such effect (data not shown).

### Co-downregulation of NF-κB and JNK Correlates With Prolonged Survival and Decreased Malignancy

**FIGURE 4:**
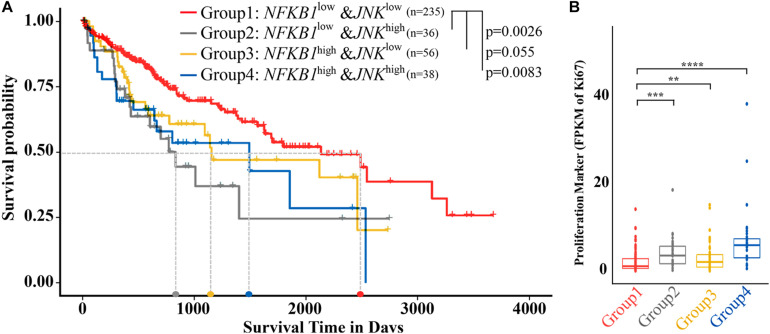
Liver cancer patients with low NF-κB and JNK expression showed a better prognosis and decreased proliferation. **(A)** Survival analysis of samples from patients with low expression of both NF-κB1 and JNK (group 1) and other types of samples. **(B)** Differential expression of Ki-67 in five types of samples. NF-κB^LOW^ and NF-κB^HIGH^ represent samples from patients whose NF-κB expression level is lower or higher, respectively, than the cutoff value of 6.18. JNK^LOW^ and JNK^HIGH^ represent samples from patients whose NF-κB expression level is lower or higher, respectively, than the cutoff value of 3.06. The light gray dotted line represents the median survival time of each group. Columns, median (*t*-test; **p < 0.01, ***p < 0.001, ****p < 0.0001).

The above data collected in cell lines and a zebrafish model suggest that the coinhibition of NF-κB and c-JUN results in preferential synergistic anti-HCC effects. Extrapolating these findings to humans, we compared patient survival across 365 individuals with HCC with different expression levels of signature genes based on the recommended parameters from the Protein Atlas database via data from TCGA (Pontén et al., 2008; Supplementary Table 5, see section “Materials and Methods”). As shown in Figure 4A, low expression of both NF-κB1 and JNK in HCC patients (NF-κB1^LOW^JNK^LOW^) was significantly associated with prolonged median survival time (50% survival probability). As determined from the patient data, the median survival time reached approximately 6.85 years (2,500 days) for the NF-κB1^LOW^JNK^LOW^ patient group and approximately 2 years (800 days), 3.3 years (1,200 days), and 4.1 years (1,500 days) for the NF-κB1^LOW^JNK^HiGH^ group, the NF-κB1^HIGH^JNK^LOW^ group, and the NF-κB1^HIGH^JNK^HIGH^ group, respectively, which increased the median survival time from 67 to 213%. Noticeably, no patient in the NF-κB1^HIGH^JNK^HIGH^ group survived longer than 7 years (2,600 days). In contrast, the survival probability of patients in the NF-κB1^LOW^JNK^LOW^ group who lived longer than 7 years was achieved at 40%. In fact, at the observation endpoint of 11 years (4,000 days), 25% of NF-κB1^LOW^JNK^LOW^ patients remained alive, demonstrating the potential value of these two targets in combating HCC development.

The Ki-67 protein has been widely used as a proliferation marker for tumor malignancy to assess the growth fraction of a cellular population. A large-scale meta-analysis provided evidence that a high Ki-67 index was closely associated with histological grade, tumor size, the number of tumor nodes, metastasis status, cirrhosis, and vein invasion in HCC patients (Luo et al., 2015). We then investigated the expression of the Ki-67 gene in the different groups (Figure 4B). Similar to the results of survival analysis, NF-κB1^LOW^JNK^LOW^ showed the lowest median Ki-67 expression, indicating the lowest malignancy potential, among the HCC patient groups, while NF-κB1^HIGH^ JNK^HIGH^ had shown the highest Ki-67 expression, suggesting the highest risk of malignancy among the groups. A significant difference in Ki-67 values was observed between the NF-κB 1^LOW^JNK^LOW^ and other groups. Consequently, compared with the other patients, HCC patients whose NF-κB and JNK are both downregulated are strongly correlated with higher overall survival (OS) and lower malignancies.

So far, the synergy inducers in HCC probed by BER and EVO have been comprehensively supported by selective inhibitors at different levels of cell line, animal model, and clinical patients. Mechanistically, coinhibition of both NF-κB and c-JUN contributes to enhancing not only antiproliferation but also antimetastasis and anti-angiogenesis *in vivo*, which may help to explain the better prognosis in HCC patients.

### Strategy of Identifying Synergy-Mediated Targets From Natural Compounds

**FIGURE 5:**
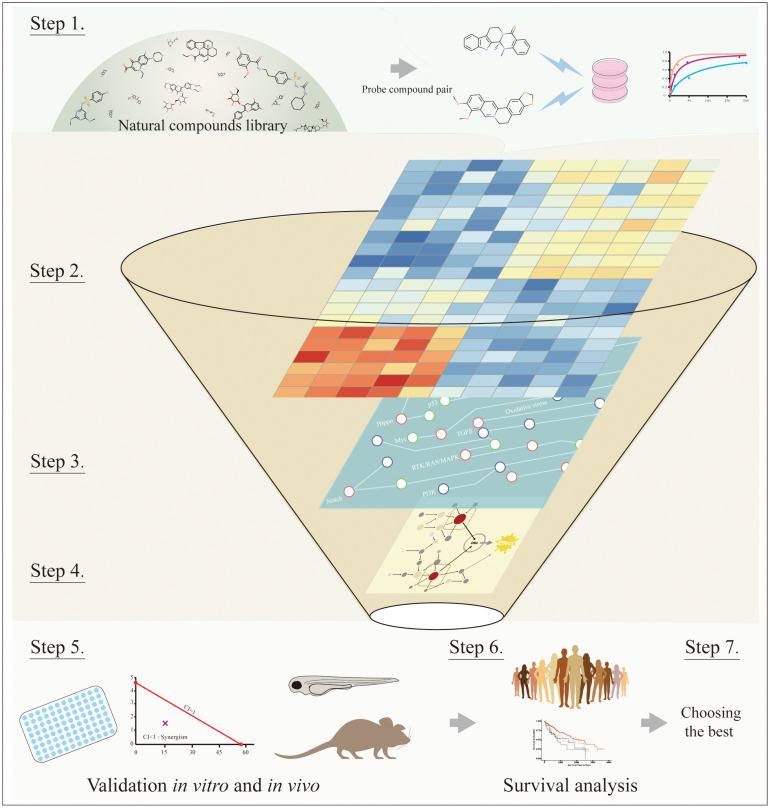
The workflow of systematic processes to identify key synergy-mediated players. Step 1: Choosing the natural compound pair from the natural compounds library. Step 2: Obtaining DEGs from transcriptomics or proteomics profiling. Step 3: Mapping DEGs to cancer signaling transduction networks. Step 4: Inferring key synergy-mediating targets among DEGs based on network topology and empirical experience. Step 5: Validating suspected targets through *in vitro* and *in vivo* experiments. Step 6: Conducting survival analysis in clinical samples. Step 7: Choosing the best candidates.

Identifying appropriate target combinations involving synergistic effects remains challenging in the context of complicated cancer signaling networks, while natural therapeutic compounds with synergistic anti-HCC effects may provide useful clues to pinpoint some candidates. Summarized from above, we here propose a reverse strategy to suggest synergy-responding inducers when potentiating effects are observed by the cotreatment. The main idea is to infer target pairs medicating drug synergy from -omics derived and signaling network, then make subsequent validation via selective inhibitors for suspected targets. The detailed workflow is shown in Figure 5: (1) choosing the natural compound pair with synergistic anticancer effects; (2) obtaining differential expressed genes/proteins (DEGs/DEPs) of cotreatment based on -omics data, such as transcriptomics or proteomics profiling; (3) mapping DEGs to cancer signaling transduction networks; (4) inferring key synergy-mediating targets among DEGs based on network topology and empirical experience; (5) deploying selective inhibitors to test the cooperative function between suspected targets through *in vitro* and *in vivo* experiments; (6) conducting survival analysis in clinical samples; and (7) choosing the best candidates.

## Discussion

Wide exploration of the targeting space is critically important to design rational combination therapies for aggressive cancers of HCC. Herein, we evaluated the feasibility to probe synergistic targets in cancer signaling networks from natural compounds in the frame of -omics analysis, network pharmacology, and experimental validation via selective inhibitors. Applying this idea, NF-κB and c-JUN were identified by BER and EVO as mediating anti-HCC effects synergistically, which were consistently supported by inhibitor validation *in vitro/vivo*, as well as clinical evidence.

Constitutive NF-κB and JNK activations were found necessitating maintaining the inflammatory and malignant microenvironment of liver cancer (Chang et al., 2009). NF-êB is well known to be widely involved in cell proliferation, apoptosis, invasion, angiogenesis, and metastasis in multiple cancers (Pikarsky et al., 2004). Recently, NF-κB inhibition was found with tumor-suppressive effect in HCC (Tsang et al., 2016) and the NF-κB inhibitor (Bay11-7082) was observed to reduce cell viability, increase ROS formation, release cytochrome c, and induce apoptosis in HepG2 cells (Ahmadian et al., 2018). Meanwhile, c-JUN acts as an oncogene promoting liver tumorigenesis to induce HCC (Trierweiler et al., 2016). Its N-terminal kinase (JNK) is known as an important signaling molecule that can transform external stimuli into a variety of cellular proliferative, invasive, and apoptotic responses, whose upregulation was found to contribute to the development of HCC (Wang and Tai, 2016). While parallel and independent, cross-talks among above pathways could cause interplay between their regulatory mechanisms, controlling numerous cellular events in the process of certain types of cancer (Nakano, 2005; Liu and Lin, 2007). In the complicated HCC networks, the single blocking of one pathway alone may not have sufficient inhibitory effects or even induce inevitable drug resistance (Gedaly et al., 2012; Xia et al., 2018), while the simultaneous targeting of two parallel or cross-talked cancer-related pathways was suggested as a significant mechanism to produce synergistic effects (Jia et al., 2009; Sun et al., 2015). Besides, in invasive HCC tissues, a positive correlation was shown between increased NF-κB, matrix metalloproteinases (MMPs), and metastasis (Zhang et al., 2013). On the other hand, HCC metastasis can be inhibited by norcantharidin through the NF-κB signaling pathway (Yeh et al., 2012), implying the pivotal role of NF-κB in HCC metastasis. In this work, it is also interesting to observe the dose-dependent angiogenesis-suppressive effects of JSH-23 through inhibiting NF-κB activity in the zebrafish model, ideal to test anti-angiogenic compounds (Howe et al., 2013).

Serving as an important source (33%) of cancer drugs developed (Newman and Cragg, 2016), natural therapeutic compounds have been reported by a body of evidence *in vitro* and *in vivo* on their potential to sensitize cancer drugs (Cote et al., 2015; Cheng et al., 2016). This coupled with their ability to inhibit some targets considered to be conventionally “undruggable,” offering a unique advantage in exploring new targeting space for combinational cancer drugs. Previous challenges in efficiently harnessing their information lay in collecting direct targets from the public domain. Strategically, the -omics change to some extent represents a dynamic profiling of compound–target interactions, containing useful information to assess anticancer effects for combinational components as well as other drug activities and responses (Palmer et al., 2019). Advances in cancer system biology enabled the integration of signaling networks, pathway profiles, and -omics change of drug treatment, which can be utilized to model the combinational effects of compound actions (Li et al., 2020) or infer cooperative partners for synergistic actions, as being demonstrated in this work.

In cancer signaling networks, empirical suggestions to infer synergy targets usually cover, but are not limited to, the following points: (1) They are truly DEGs/DEPs in the cotreatment compared to blank controls. (2) They should be consistently regulated in both mono- and cotreatment. (3) They are usually located in key nodes of parallel, or redundant, or cross-talked, or compensatory pathways within certain network topology distances (Jia et al., 2009; Sun et al., 2015; Cheng et al., 2019).

It is cautioned that the synergistic level of different natural combinations may be influenced by genetic variations of the cancer subtype, the dosage of each ingredient, and even drug scheduling (Bansal et al., 2014). Many synergy-responding elements are much more complicated than those mechanisms inferred so far, and their activities are highly dynamic. Therefore, besides NF-κB and c-JUN signaling, additional combinations might also exist in HCC following the mechanism of updating of drug synergy summarized. So the use of this probing strategy should be more appropriately viewed as a start to a more comprehensive optimized screening of synergy inducers at large-scale and system levels.

## Data Availability Statement

The datasets presented in this study can be found in online repositories. The names of the repository/repositories and accession number(s) can be found in the article/ Supplementary Material.

## Author Contributions

JG and ZY contributed to the overall design. ZW, CM, ZS, and JG performed the wet experiments. RC and XZ contributed to the experiment of metabolism. JG, ZY, CM, and ZS analyzed and interpreted the results. JG, ZY, and ZW wrote the manuscript. XZ, KT, JF, and ZC modified the manuscript. ZC, JF, and KT supervised the project. All authors read, critically reviewed, and approved the final manuscript.

## Conflict of Interest

ZW was employed by company Shanghai Model Organisms Center, Inc. The remaining authors declare that the research was conducted in the absence of any commercial or financial relationships that could be construed as a potential conflict of interest.

## Publisher’s Note

All claims expressed in this article are solely those of the authors and do not necessarily represent those of their affiliated organizations, or those of the publisher, the editors and the reviewers. Any product that may be evaluated in this article, or claim that may be made by its manufacturer, is not guaranteed or endorsed by the publisher.

## Footnotes

1
http://www.cellbank.org.cn/
