# Supplementary material for: Probing Synergistic Targets by Natural Compounds for Hepatocellular Carcinoma
Source: Front Cell Dev Biol. 2021 Jul 28;9:715762. doi: 10.3389/fcell.2021.715762 (PMC8355820; doi:10.3389/fcell.2021.715762)
Supplement: Supplementary file 1 [file Data_Sheet_1.pdf]

Supplementary information

Supplementary Figures

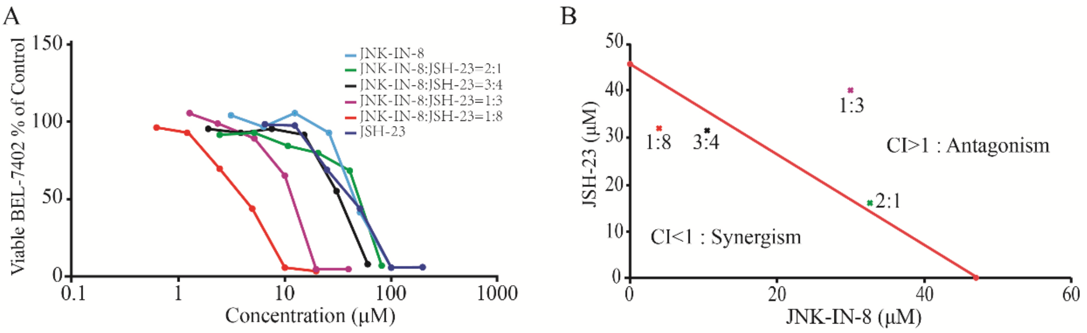

Figure S1. Co-inhibitors suppresses cell growth on BEL-7402 cells. **(A)** The dose-response curve of each single inhibitor or their combinations. For combinations, the x-axis represents the concentration of JNK-IN-8. **(B)** Isobologram for the combination of JNK-IN-8 and JSH-23 in BEL-7402 cells. The red line represents the intercept line of the IC50 for JNK-IN-8 and JSH-23 alone, and the points stand for the IC50 of the two inhibitors at the different dose ratios of 1:8, 3:4, 1:3, 2:1. CI <1, =1, and >1 indicate synergism, additive effect, and antagonism, respectively.

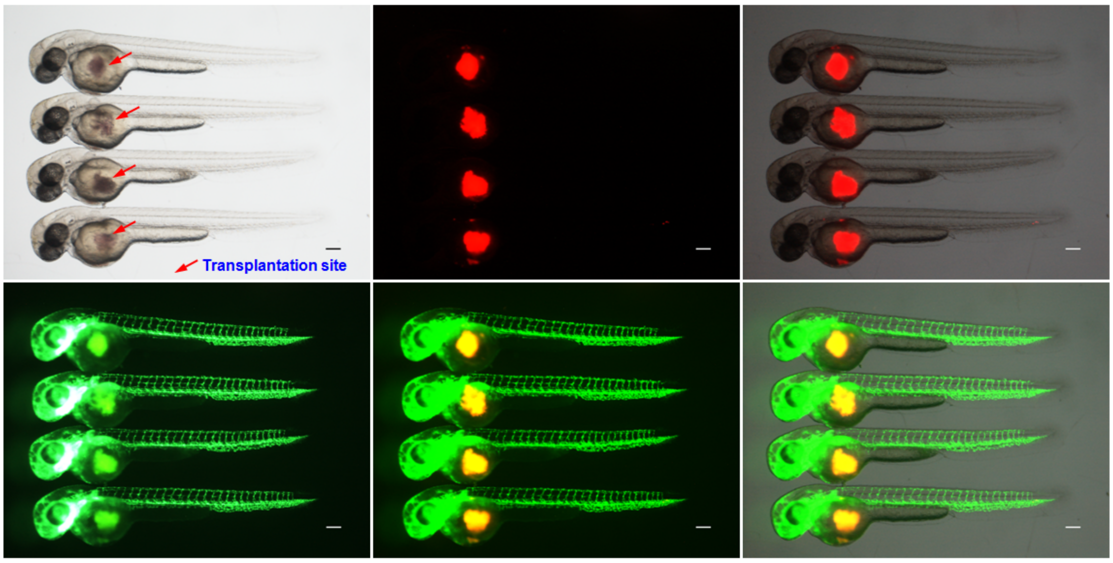

Figure S2. Establishment of zebrafish model in vivo. DiI-stained human BEL-7402 cells were successfully grafted into the yolk sac of zebrafish embryo at 2 days post fertilization (dpf) without immunosuppressant treatment. Approximately 200 cells were injected into the yolk sac and assessed by fluorescence microscopy. dpi, days post-injection. Scale bar, 100 μm.

A

#Animal Number: N=20/each group		JSH-23 (μM)										
Endpoints	Control	0.1	1	10	20	25	30	40	50	75	100	200
Pericardial oedema	0	0	0	6/20	10/20	5/20	4/20					
Mortality rate	0	0	0	0	10/20	15/20	16/20	20/20	20/20	20/20	20/20	20/20

B

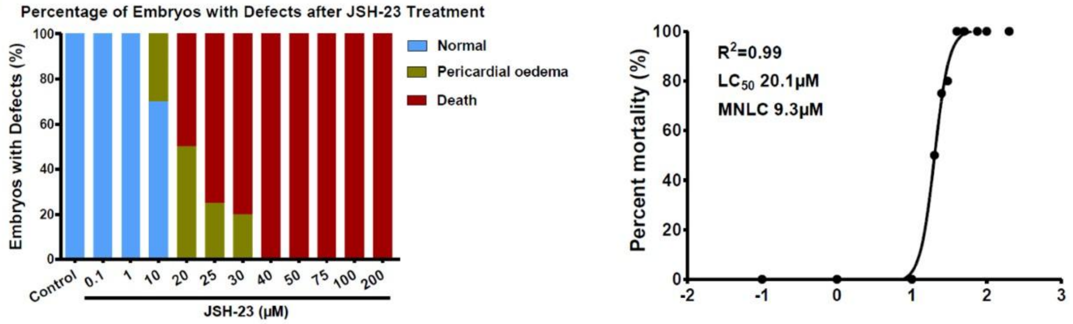

C

#Animal Number: N=20/each group		JNK-IN-8 (μM)									
Endpoints	Control	0.1	1	10	25	40	50	60	75	100	250	500
Mortality rate	0	0	0	0	0	0	3/20	14/20	16/20	20/20	20/20	20/20

D

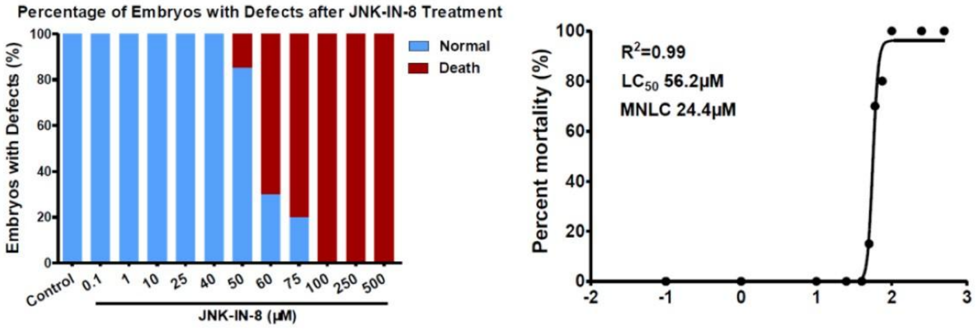

E

Group	Treatment	Route	Number of Animals
1	Vehicle Control	Soaking	20
2	JSH-23 (5μM)	Soaking	20
3	JSH-23 (10μM)	Soaking	20
4	JNK-IN-8 (10μM)	Soaking	20
5	JNK-IN-8 (25μM)	Soaking	20
6	JSH-23 (10μM) + JNK-IN-8 (25μM)	Soaking	20
7	JSH-23 (10μM) + JNK-IN-8 (10μM)	Soaking	20
8	JSH-23 (10μM) + JNK-IN-8 (4μM)	Soaking	20
9	JSH-23 (10μM) + JNK-IN-8 (2μM)	Soaking	20
10	JSH-23 (5μM) + JNK-IN-8 (25μM)	Soaking	20
11	JSH-23 (5μM) + JNK-IN-8 (10μM)	Soaking	20
12	JSH-23 (5μM) + JNK-IN-8 (4μM)	Soaking	20
13	JSH-23 (5μM) + JNK-IN-8 (2μM)	Soaking	20

F

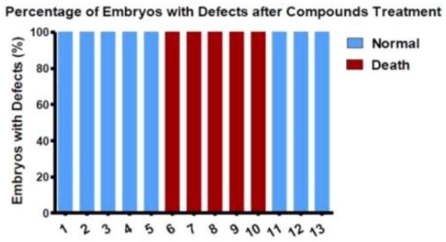

Figure S3. The evaluation of maximum non-lethal concentration (MNLC) and LC50 determination, as well as safety evaluation of JSH-23 and JNK-IN-8. **(A)** Statistic number of animal situations observed after JSH-23 treatment. **(B)** Graphic representation of the data from A. **(C)** Statistic number of animal situations observed after JNK-IN-8 treatment. **(D)** Graphic representation of the data from C. **(E)** The tested combinations of JSH-23 and JNK-IN-8. **(F)** The safety evaluation of tested combinations. All compounds were dissolved in 0.1% DMSO (in fish water). Vehicle Control: in 0.1% DMSO (in fish water).

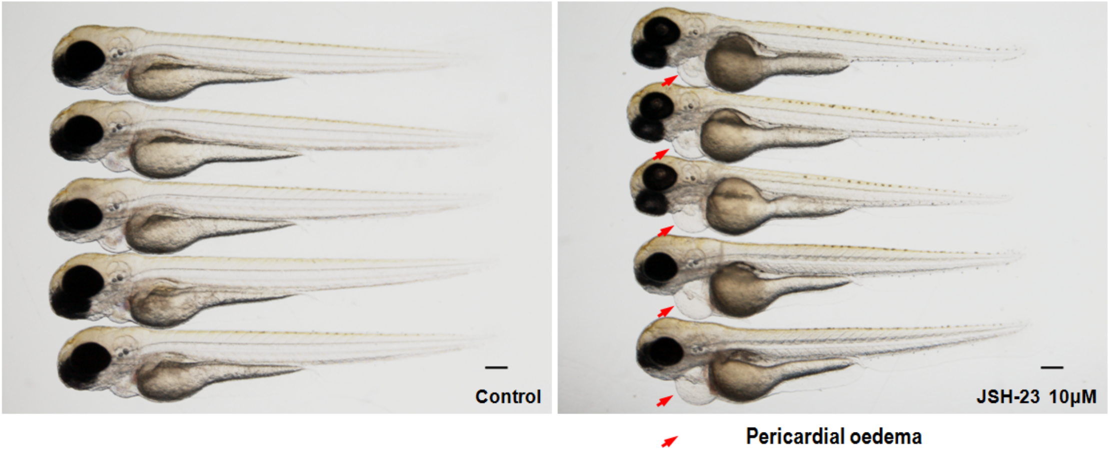

Figure S4. pericardial oedema for zebrafish embryos after JSH-23 treatment. Scale bar, 100 μm.
